# Estradiol, progesterone, testosterone profiles in human follicular fluid and cultured granulosa cells from luteinized pre-ovulatory follicles

**DOI:** 10.1186/1477-7827-8-117

**Published:** 2010-10-11

**Authors:** Xuesong Wen, Dong Li, Amanda J Tozer, Suzanne M Docherty, Ray K Iles

**Affiliations:** 1 Biomedical Sciences, School of Health and Social Science, Middlesex University, The Burroughs, NW4 4BT, UK; 2 The Williamson Laboratory, St Bartholomew's and the Royal London School of Medicine and Dentistry, Queen Mary University of London, London EC1A 7BE, UK; 3 The 1st Affiliated Hospital of Harbin Medical University, Harbin, 150001, China

## Abstract

**Background:**

The production of sex steroids by follicular cells is proposed to be influenced by the maturity of the incumbent oocyte. Thus steroid levels may reflect suitability of an oocyte for IVF. We examined follicular fluids and granulosa cell production of steroid from IVF patients in order to test the relationship between steroid levels and fertilization.

**Methods:**

Follicular fluid and granulosa cells were extracted from 206 follicles of 35 women undergoing controlled ovarian stimulation. Follicular fluid was assayed for estradiol, progesterone and testosterone. Granulosa cells were cultured from individual follicles and their culture media assayed for production of these hormones after 24 hrs in vitro. Levels of steroids were correlated with follicular diameter, oocyte recovery and subsequent fertilization.

**Results:**

Follicular fluid levels of progesterone were 6100 times higher than that of estradiol, and 16,900 times higher that of testosterone. Despite the size of follicle triggered after controlled luteinisation, the levels of progesterone and testosterone were maintained at relatively constant levels (median 98.1 micromoles/L for progesterone, and 5.8 nanomoles/L for testosterone). However, estradiol levels were slightly lower in the larger follicles (follicular diameter 10-15 mm, median 25.3 nanomoles/L; follicles > = 15 mm, median 15.1 nanomoles/L; linear correlation r = -0.47, p < 0.0001). With respect to oocyte recovery, no steroid showed a significant association in follicular fluid levels. Similarly no difference in follicular fluid steroid levels was found for those oocytes that did or did not fertilize. Significant quantities of progesterone were produced by the granulosa cells but production was constant regardless of the size of follicle from which the cells originated. Estradiol levels were only detectable in 10 of 121 cultures examined, and testosterone in none. Interestingly, when an oocyte was present follicular estradiol levels correlated with progesterone levels. However, when absent, follicular estradiol levels correlated with testosterone levels but not with progesterone.

**Conclusions:**

The principle steroid product of luteinized pre-ovulatory granulosa is progesterone, a differentiation triggered by the gonadotropin surge. However, absolute steroid levels are associated with follicular size, not oocyte maturation/ability to fertilize.

## Background

Oocyte maturation is a complex process that includes the re-initiation and completion of the first meiotic division and subsequent progression to metaphase II, as well as nurse cell preparation of the oocyte cytoplasm. As the whole process of nuclear and cytoplasmic change is crucial for fertilization and early embryo development, investigations have concentrated on the composition of the intercellular environment of this process, the follicular fluid.

Estradiol, progesterone and testosterone are the main steroid hormones that play essential roles during the follicular and luteal phases of the menstrual cycle. However other cytokines, such as inhibins, activins, insulin growth factor-2, insulin growth factor binding proteins, tumour growth factor-β and endothelial growth factor, have been measured in follicular fluid and correlated with oocyte maturation [1-4]. Nevertheless, only serum concentrations of estradiol and follicular size are routinely used for monitoring follicular development and oocyte maturity during ovarian induction for assisted reproduction [5-8].

Despite this, there are still inconsistencies in the literature regarding estradiol and the other steroid hormone concentrations in follicular fluids, and also in granulosa cell cultures. There is even less information on steroid secretion profiles of granulosa cells cultured from individual follicle. Ryan and Smith carried out the first study of the biosynthesis capability of the human ovarian follicle in 1961 [9,10]. They showed that apart from estradiol and estrone, there were also some androgens and progesterone produced. However, this experiment was compromised by the fact that they incubated the cells in the presence of progesterone because a prevailing hypothesis claimed that this was a necessary extrinsic precursor of granulosa/theca estrogen synthesis. In an earlier study on rat follicles, Falck [11] had noted that both theca and granulosa cells that were necessary for oestrogen biosynthesis result from an interaction of the two cell types. However, Channing and Coudert [12] showed that monkey theca cells alone were able to produce normal amount of estrogens. Thus they concluded that theca cells were the main producers of estradiol rather than granulosa cells. This is completely different from the accepted 'two-cell, two-gonadotropin' theory of follicular steroidogenesis which is found in multiple texts. According to this theory, granulosa cells are the main source of estradiol production, which results from conversion of theca cell-derived androgens [13,14,2]. This is largely based on reports that aromatization is stimulated by FSH, whose activity can only be activated by binding to its receptor on granulosa cells [2]. Indeed, it is commonly accepted that theca cells produce progesterone rather than estradiol due to this lack of aromatase (CYP19) activity [15,2].

This study set out to investigate the levels of estradiol, progesterone and testosterone found in individual follicular fluid from stimulated cycles and their granulosa cell cultures after oocyte retrieval. The principal aim was to investigate steroid hormone levels in relationship to follicular diameter, oocyte presence and subsequent fertilization. However, this also allowed us to investigate steroidogenesis within the ovarian-follicular environment following stimulation for IVF, a situation akin to a follicle after the gonadotropin surge.

## Methods

### Patients

**Figure 1:**
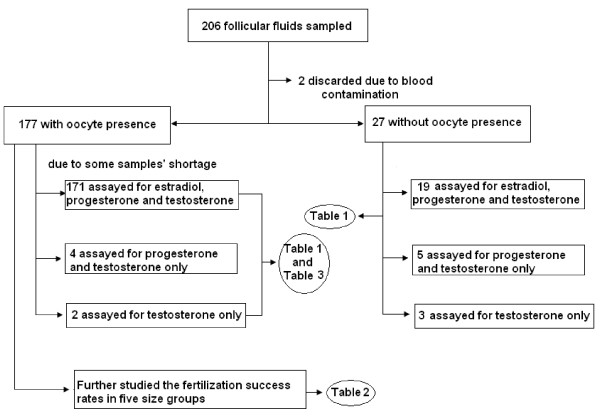
**Samples and sample analysis**. Consort-type diagram to show the sample distribution and numbers in the study analysis.

35 patients who undertook *In Vitro *Fertilization/Intracytoplasmic Sperm Injection at the Fertility Unit of St Bartholomew's Hospital from 1999 to 2001 were recruited into this study. The ages of patients varied from 29 to 38 years old, and the follicular size varied from 10.2 mm to 35 mm. The total number of the follicles sampled was 206; 4 to 6 follicles per patient. The causes of infertility in this patient cohort were male, tubal or unexplained factors. Patients with endometriosis and polycystic ovarian syndrome were excluded from this study, as ovarian steroid hormone production is known to be altered in these conditions. Due to blood contamination, limited sample volume and lack of follow-up information, the final distribution of sample numbers between oocyte recovery, fertilization outcome and steroid analysis is shown in figure 1.

Written consent was obtained from each patient, and this study was approved by the East London and The City Health Authority Research Ethics Committee (study number: P/98/222).

### Sample collection

Sample collection was 36 hours after subcutaneous administration of human chronic gonadotropin [10,000 IU (Profasi, Serono)], and when at least three follicles in each patient had reached 18 mm in diameter as measured by ultrasound scan (Hitachi, model EUB-525, Northants, UK). Individual follicles were randomly selected prior to aspiration and measured in two dimensions by transvaginal ultrasound in order to obtain a mean diameter. The same operator performed all measurements and aspirations. Following identification of a suitable follicle, the follicle was gently pierced using a double lumen needle and aspirated, allowing the follicle to collapse slowly around the needle. The follicle was then flushed with 4.5 ml of heparinised saline (3×1.5 ml automated flushes) and aspirated again. Heavily blood stained aspirates were discarded and a further follicle was measured and aspirated if appropriate. A detailed record was kept of the volume of fluid aspirated per individual follicle prior to washing. Following examination of the follicular fluid, it was placed in a sterile tube and the presence of an oocyte was noted and recorded. If an oocyte was not retrieved, the follicle was flushed again with an additional 3 ml heparinised saline. If an oocyte was still not obtained, the follicle was recorded as having no oocyte retrieved. Then the follicular fluids were taken immediately to the Williamson Laboratory. Each tube was centrifuged at 200×*g *for 10 minutes to precipitate harvested granulosa cells. Follicular fluid supernatant was aspirated, divided into aliquots, and frozen at -20°C for subsequent hormone analysis. The fertilization outcome was also recorded according to the appearance of two pro-nuclei after incubating in cultured medium after 48 hours.

### Isolation and culture of granulosa cells from individual follicles

Harvested follicular fluid cell pellets were resuspended in 4 ml of medium. This consisted of RPMI-1640 with glutamine and NaHCO_3 _[Sigma, Poole, UK] supplemented with 10%v/v fetal calf serum (FCS) [GibcoBRL, Paisley, UK] and 1%v/v of an antibiotic stock solution containing 10,000 μg/ml Penicillin G sodium and 25 μg/ml Streptomycin [GibcoBRL, Paisley, UK]. After washing and re-centrifugation at 200×*g *for 10 minutes, cells were resuspended in 5 ml of medium containing 0.2% hyaluronidase (80IU/ml. Medicult, UK) for cell dispersion, and then incubated at 37°C for 30 minutes. After this time, the sample was again centrifuged at 200×g for 10 minutes and the cell pellet resuspended with 2 ml of medium and layered over 4 ml of 50% Percoll [Pharmacia, Amersham, UK]. This was centrifuged at 250×*g *for 20 minutes to separate the luteinized granulosa cell buffy coat from any contaminating erythrocytes. The granulosa cells were removed using a pipette and washed with 8 ml of medium. After a final centrifugation at 250×*g *for 10 minutes the pellet was resuspended and the cell number was determined using a haemocytometer. Cells were then plated, in triplicate wells, at a concentration of 10,000 cells per well in 24 well culture plates (Corning, NY, USA) and incubated at 37°C in a 95% air, 5% CO_2 _humidified environment for 24 hours and the cell free supernatant media was then collected and stored at -20°C for later analysis.

### Steroid measurement

Steroid hormone levels of progesterone, estradiol and testosterone were measured in follicular fluid and culture media by radioimmunoassay (RIA). These assays were carried out by commercial kits provided by Skybio Limited (Bedford, UK).

Testosterone - The minimum detection of this essay was 0.2 nmols/L, and its standard range was from 0.7 nmols/L to 69 nmols/L. The cross reaction of this assay is reported to be lower than 0.01% for androstenedione, dihydroxytestosterone, estrone, estradiol-17β and progesterone. Quality control data from repeat assays showed < 10% CV.

Estradiol - The minimal detectable concentration was 25 pmols/L, and its standard range was from 37 pmols/L to 11,100 pmols/L. The cross reactivity of this assay is reported to be: estrone < 6.2%, estradiol < 1.5%, cholesterol, DHEA, 17α-Hydroxyprogesterone, pregnenolone, progesterone and testosterone all lower than 0.01%. Quality control data from repeat assays showed < 15% CV.

Progesterone- The minimum detectable concentration was 4 nmols/L, and its standard range was from 5 nmols/L to 80 nmols/L. Consequently samples needed to be diluted between 10 and 100 fold. The cross reactivity of this assay is reported to be: 17α-Hydroxyprogesterone < 0.5%, pregnenolone < 0.3%, testosterone, estradiol-17β, cholesterol all < 0.001%. Quality control data from repeat assays showed < 8% CV.

### Statistical analysis

Data was tabulated in Excel and imported in to a data analysis software StatsDirect package (StatsDirect Ltd, Cheshire, UK) and a p < 0.05 was taken as statistical significance. Graphical analysis was initially conducted as scatter plots where the concentrations and calculated total content of the steroid hormones were plotted against its individual follicular diameter and correlations tested. Distribution analysis revealed that the data generated had a non-Gaussian distribution; consequently, non-parametric statistical description and statistics have been used. Spearman rank correlation analysis was used to compare the inter-relationship between the examined steroids.

## Results

### Steroid levels in follicular fluid

**Figure 2:**
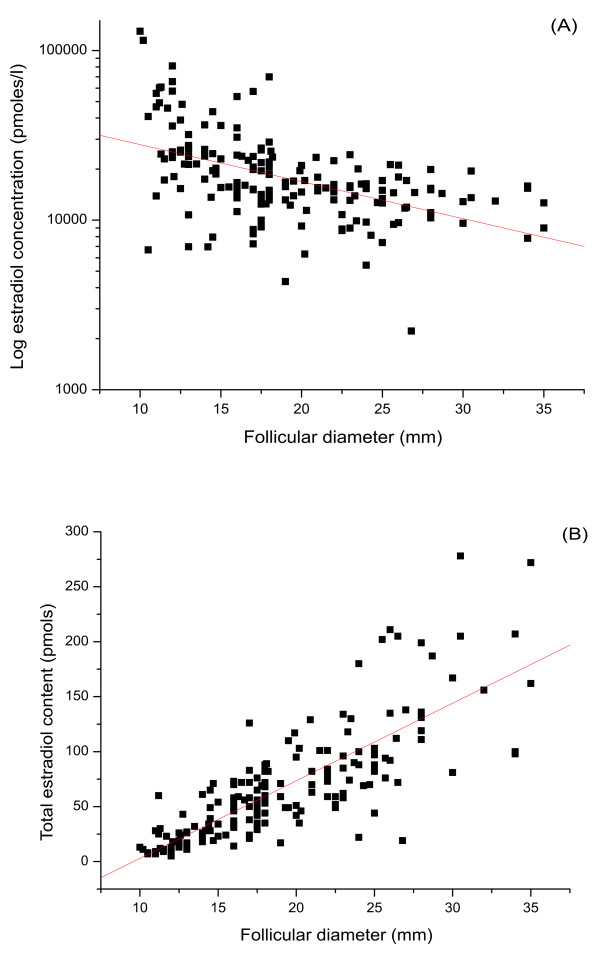
**Follicular estradiol**. Scatter plots to show the correlation of estradiol concentration (pmoles/L) (a) and total content (pmoles) (b) with follicular diameter (mm). Estradiol concentration significantly decreased with follicular size increase (2a: r = 0.5, p < 0.001); while estradiol total content increased dramatically when corrected by follicular volume (2b: r = 0.79, p < 0.001).

For all follicular fluid samples, estradiol concentrations varied from 2.2 - 130.0 nmoles/L (median = 16.1 nmoles/L). Levels significantly decreased as follicular size increased (figure 2a: r = -0.47, p < 0.0001). A sharp decrease in estradiol concentration was indicated between 10 and 20 mm follicle diameter, and a plateau concentration was seen for follicles ≥20 mm in diameter. However, when corrected by follicular volume, the total follicular content of estradiol increased with follicular diameter (figure 2b: r = 0.79, p < 0.0001).

**Figure 3:**
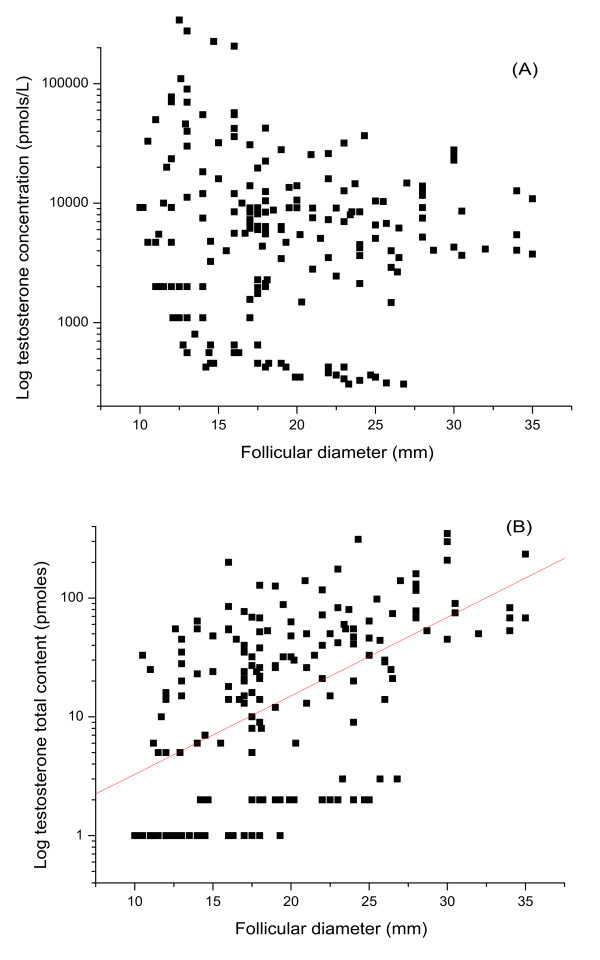
**Follicular testosterone**. Scatter plots to show the correlation of testosterone concentration (pmoles/L) (a) and total content (pmoles) (b) with follicular diameter (mm). The concentration of testosterone did not correlate with follicular diameter (3a), but total content increased with the follicular size (3b) (r = 0.52, p < 0.001).

**Figure 4:**
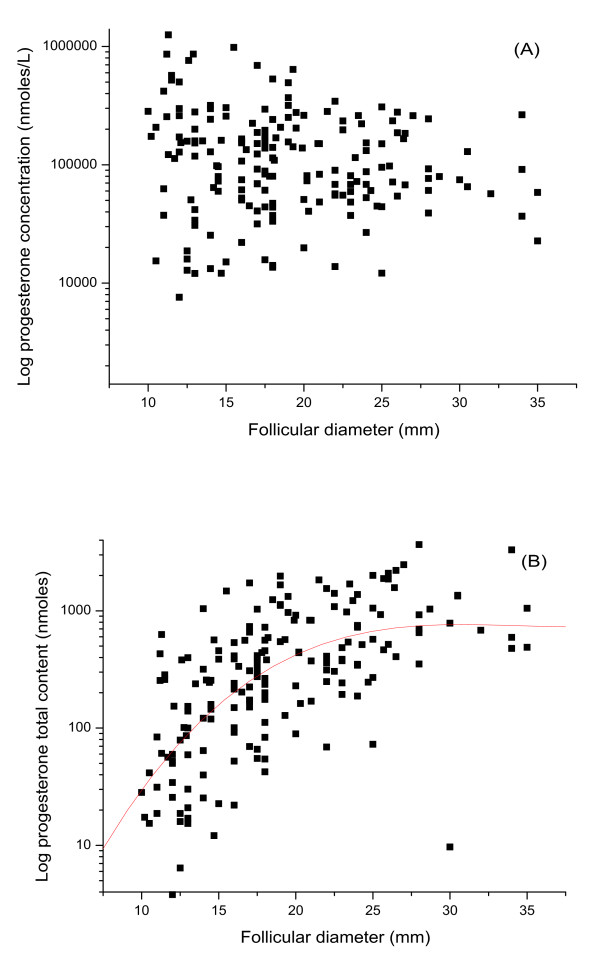
**Follicular progesterone**. Scatter plots to show the correlation of progesterone concentration (nmoles/L) (a) and total content (nmoles) (b) with the follicular diameter (mm). The concentration slightly decreased (4a: r = -0.15, p < 0.05) but its total content increased with follicular size (4b: r = 0.64, p < 0.0001).

Testosterone concentration varied from 0.3-110.0 nmoles/L (median = 5.8 nmoles/L). The general trend was for testosterone concentration to remain constant despite follicular size (figure 3a). Consistent with this finding was that total testosterone content within each individual follicle increased with the size of the follicle (figure 3b: r = 0.52, p < 0.0001). Progesterone concentration varied from 1.1-1255.0 μmoles/L (median = 98.1 μmoles/L). Although the general trend was for progesterone concentration to remain constant despite follicular size (figure 4a), the total progesterone content of the follicles correspondingly increased (figure 4b: r = 0.64, p < 0.0001).

### *In vitro *secretion of steroids by cultured luteinized granulosa

All three steroid hormone levels were measured at 24 hours in culture media taken from granulosa cells isolated from individual follicles, and grown in triplicate. Testosterone was not detected in any of the 110 granulosa cell cultures examined. Only 10 out of 121 granulosa cell cultures examined contained detectable levels of estradiol production (15, 15, 16, 65, 77, 88, 108, 150, 152, 489 pmoles/L/10,000 cells/24 h). Significant levels of progesterone production were detected in all 104 granulosa cell cultures examined (median value was 409 nmoles/L/10,000 cells/24 h; inter-quartile range 247-925 nmoles/L/10,000 cells/24 h). Progesterone production by granulosa cells was relatively constant and did not correlate with the diameter of the follicle from which they originated (data not shown).

### Relationship of steroid hormone levels with oocyte presence and fertility outcome

There was no significant difference in progesterone production at 24 hours in cultured medium between the groups with or without an oocyte present. Similarly, the follicular fluid concentration of all three steroids showed no statistically significant difference between follicles from which an oocyte was or was not recovered. This was despite a general trend of lower median levels of testosterone in the group from which an oocyte was recovered (see table 1). For the recovered oocytes, there was no significant difference in follicular fluid concentration of progesterone, estradiol or testosterone (see table 1). When grouped into size bins increasing by 3 mm diameter up to 25 mm (equalized to 5 groups representing ascending 20^th ^centiles of the study populations), it became clear that fertilization rates increased dramatically for follicles of greater than 13 mm (10-13 mm diameter- 30%, 14-17 mm - 62%). However, the fertilisation rates slowly declined such that the very large follicles (> 25 mm) had a recovered oocyte fertilization rate similar to that of small follicles (< = 13 mm) (see table 2).

**Table 1:** Descriptive and statistical analysis of follicular fluid concentrations of estradiol, testosterone and progesterone from follicles grouped by oocyte presence and its subsequent fertilization outcome (comparisons were by the Mann-Whitney U test).

Group	Estradiol Concentration (nmoles/l) Median (25^th^-75^th ^Centile)	Progesterone Concentration (μmoles/l) Median (25^th^-75^th ^Centile)	Testosterone Concentration (nmoles/l) Median (25^th^-75^th ^Centile)
**Oocyte**	15.6 (13.6-22.5)	121.9 (68.9-241.3)	4.7 (0.9-12.7)
**No oocyte**	19.0 (12.9-51.3)	153.7 (82.2-300.2)	9.1 (0.9-24.6)
**p value**	p = 0.33	p = 0.38	p = 0.25
**Fertilized**	15.3 (11.2-23.2)	109.1 (68.9-241.3)	5.5 (1.5-13.1)
**Unfertilized**	17.2 (13.7-23.8)	131.2 (70.7-205.2)	6.2 (1.0-18.0)
**p value**	p = 0.36	p = 0.72	p = 0.97

**Table 2:** Descriptive analysis on fertilization success rates and follicular fluid steroid concentrations of follicles grouped according to their ascending follicular size (approximately 3 mm incremental bins and representing ~20 centiles of the size ranked study population).

Follicular size (mm)	n	Percentage fertilized	Progesterone concentration (μmoles/l) NF FERT		Estradiol concentration (nmoles/l) NF FERT		Testosterone concentration (nmoles/l) NF FERT	
10-13	33	30%	128.1 (34.0-282.6)	206.9 (104.3-456.7)	25.9 (19.7-52.9)	27.2 (22.5-50.9)	9.2 (2.0-32.3)	3.4 (2.0-42.5)
14-17	37	62%	142.6 (51.8-159.5)	91.8 (59.7-187.6)	23.7 (17.7-30.5)	15.9 (13.6-23.2)	1.1 0.58-12.4)	8.4 (3.3-18.3)
18-21	45	56%	159.9 (81.3-248.1)	88.4 (42.1-155.7)	15.5 (12.8-18.2)	15.4 (13.0-22.4)	6.2 (0.93-9.1)	5.6 (2.1-9.8)
22-25	32	53%	72.1 (55.3-197.3)	68.3 (48.5-122.7)	14.4 (9.9-16.3)	13.4 (9.0-15.9)	5.1 (0.43-16.0)	3.5 (0.37-8.5)
> 25	30	40%	79.5 (56.9-184.3)	76.5 (58.4-235.2)	14.4 (11.8-17.1)	13.1 (9.7-15.3)	4.2 (3.4-11.9)	8.9 (4.2-12.5)

N- the number of samples; NF- follicles containing an oocyte that failed to fertilize; FERT- follicles containing an oocyte that fertilized.

Examining the inter-correlation of the three steroids showed interesting results (table 3): When an oocyte was present, estradiol and progesterone levels always strongly correlated with each other; however, estradiol levels did not correlate with testosterone. When an oocyte was not recovered, progesterone levels did not correlate with estradiol and testosterone, but estradiol levels did correlate with testosterone (table 3). Progesterone and testosterone did not correlate when the oocyte was fertilized, but correlated when the oocyte failed to fertilize.

**Table 3:** Spears rank correlation analysis for the interrelationship between estradiol, progesterone and testosterone concentrations in follicular fluids from three groups: no oocyte presence, with a fertilized oocyte and with an unfertilized oocyte.

Group	Parameter	Progesterone	Testosterone	Estradiol
No oocyte	Progesterone	-	Rho = -0.18	Rho = 0.37
	Testosterone	p = 0.48	-	**Rho = 0.54**
	Estradiol	p = 0.14	**p = 0.02**	-
Oocyte fertilized	Progesterone	-	Rho = 0.2	**Rho = 0.49**
	Testosterone	p = 0.25	-	Rho = 0.15
	Estradiol	**p = 0.003**	p = 0.4	-
Oocyte non-ferilized	Progesterone	-	Rho = 0.35	**Rho = 0.48**
	Testosterone	p = 0.049	-	Rho = 0.13
	Estradiol	**p = 0.006**	p = 0.46	-

## Discussion

### Human granulosa cell biosynthesis of steroid hormone

Using animal cell culture systems it was proposed that both theca and granulosa cells are necessary for estrogen biosynthesis, and that the major source of this was the granulosa cells [11]. As early as the 1960s, Ryan and co-workers suggested that granulosa cells are the main producer of progesterone, not estradiol [9,10,16]. Later on, work on monkey ovarian tissues claimed that estradiol was produced solely by theca cells [12]. According to the accepted two-cell estrogen synthesis interdependence hypothesis, the principle source of estradiol is believed to be the granulosa whereby androgens from developing follicles - derived exclusively from theca cells - are transported to the granulosa and aromatized to estradiol [17].

**Figure 5:**
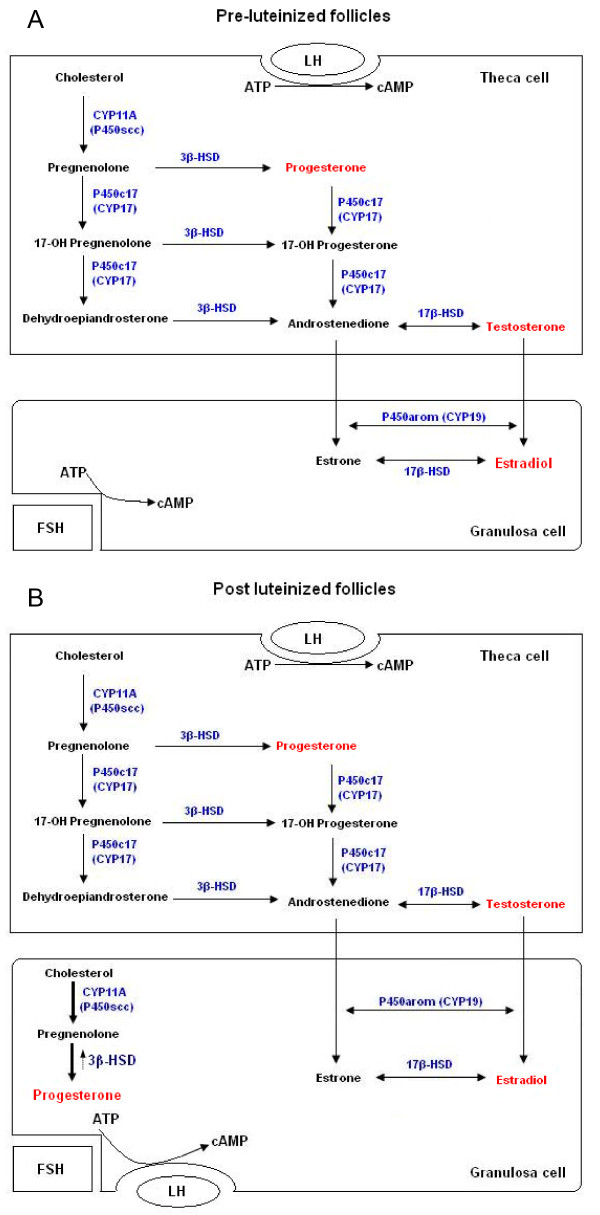
**Ovarian follicular steroid synthesis**. Diagramatic representation of the steroidogenic pathways in human pre-luteinized follicles (A) and post luteinized follicles (B), demonstrating granulosa dependence on theca cell testosterone steroidogenesis in pre-luteinized follicles and the *de novo *synthesis of progesterone from cholesterol due to induction/up-regulation of CYP11A and 3βHSD after the gonadotropin surge in post luteinized follicles.

Our follicular fluid analysis demonstrated the presence of both estradiol and testosterone in the environment of the granulosa cells when harvested. When cultured for 24 hours and the media collected, testosterone was not detected. Estradiol, although abundant in the follicular fluids, was only detected in 10 out of 121 samples. This is consistent with theca cells being the sole source of androgens, as they are the only ovarian tissue to express the enzyme P450c17/CYp17, which is responsible for converting C21 steroids (progestrogens) to C19 steroids (androgens) [18,19]. Immunohistochemical examinations of porcine follicles also showed the lack of immune-enzymatic activity of P450c17/CYP17, but highly immunodetectable levels of P450arom/CYP19 in granulosa cells [20]. Therefore, cultured granulosa cells would not be expected to synthesize estradiol unless the precursor testosterone was added. In our study, estradiol was detected in 8% of total granulosa cultured samples at low levels, which can be explained by the low levels of testosterone present in the cultures. As for the high levels of progesterone being synthesized by cultured granulosa, this can only be a consequence of up-regulated 3βHSD enzymatic activity, which is induced after the gonadotropin surge. Therefore, our findings on steroid production from post luteinized cultured granulosa provide a confirmation of steroidogenic enzymes expression changes to the "two cells, two gonadotropins" model following the LH surge:

The theca is a very thin layer of cells limited to, and defining, the peripheral boundary of the follicle. These cells express CYP17 as well as 3β-HSD such that further metabolism of either pregnenolone or progesterone (via 17-OH-pregnenolone and 17-OH-pregesterone) to androgens occurs. Thereafter, androgens can diffuse across the basal lamina into granulosa cells where they can be metabolized to estrogens, since these cells specifically express CYP19/aromatase [21] (see figure 5A).

In our study, granulosa cell cultured samples were collected after 36 hours of hCG treatment and pre-oocyte retrieval, i.e. the cells were luteinized but pre-ovulatory granulosa cells. Steroidogenic ability changes under the influence of gonadotropin surge. Two previous *in vitro *studies of cultured granulosa cells in rats also showed the increased levels of mRNA, protein and emzymatic activity of 3β-HSD after treatment with FSH [22,23]. Pelletier *et al *have shown that 3β-HSD was detected on both theca and granulosa cells from pre-ovulatory follicles [18]. Here we have shown high levels of progesterone detected in follicular fluids and also in 24-hour granulosa cultures; this suggests that 3βHSD is up-regulated in the luteinized granulosa cells and, since CYP17 is not expressed in this tissue, no further metabolism to androgens occurs, with progesterone becoming the major end steroid product (see figure 5B).

Post-luteinisation (which is synonymous with ovulation), the LH/FSH surge induces or up-regulates CYP11A and 3β-HSD expression by granulosa cells such that this is now the dominant pathway and source of progesterone.

Furthermore, the important enzyme catalyzing the crucial step for the formation of either DHEA or progesterone has been shown to have two types of isoenzymes, type 1 and type 2 [24]. The specific tissue and cellular expression and substrate specificity provide to both theca and granulosa cells unique and independent mechanisms to control the levels of intracellular active steroids [25,26]. Luteinisation may induce differential expression of the two isoforms in the two cell types.

The interrelationships among the three steroids may also reveal the maturation of granulosa tissue after the gonadotropin surge (table 3). For example, in follicles from which an oocyte was not recovered, testosterone levels correlated with estradiol; perhaps this reflects the lack of granulosa maturation which requires the presence of the oocyte. When an oocyte was recovered and subsequently fertilized, estradiol correlated with progesterone levels but not testosterone.

### Follicular fluid steroid hormone profiles - oocyte presence and subsequent fertilization

The relationship between follicular size and oocyte maturation has been debated for some time. Eppig *et al *[27] claimed that oocyte developmental competence - as shown by the ability to undergo fertilization and develop to blastocyst stage - was independent of both the size of follicle and the size of oocyte. However, the human oocyte has a size-dependent ability to resume meiosis and complete maturation: larger oocytes, or the oocytes taken from larger follicles, have a higher meiotic maturation rate [28-30]. Furthermore, McNatty *et al *[31] demonstrated that the hormone synthesising ability of granulosa cells was also dependent on the size of follicle from which they originated, principally in their aromatizing ability.

The fertilization and 2/4 cell blastocyst formation rate is lower for oocytes recovered from follicles of less than < 10 mm [32-34]. Indeed, empirical practice has shown that follicles of 18 mm are favored in assisted reproduction, and data now suggests that 14 mm is the lower end cut-off size that reflects follicular/oocyte maturity [35]. Serum estradiol is often used as a marker of follicular development [5-7,36] and the steroidogenic activity of the incumbent granulosa changes with follicular maturation. Some studies have suggested that luteinized pre-ovulatory follicular fluid levels of progesterone, estradiol, testosterone - and even the ratio between estradiol and testosterone - is a better indicator of oocyte maturity than follicular size alone [37-40].

Unfortunately, most of these studies showing a significant correlation have grouped follicles into mature and immature (sometimes intermediate) by criteria based on an arbitrary follicle size cut-off. This will bias results: oocyte maturity is a function of follicular size, whilst steroid production by the follicle is also a function of follicular mass. If follicles are split simply into large (mature) or small (immature), it is inevitable that higher levels of steroids will be found in those follicles from which there was a higher rate of fertilization. Any relationship between fertilization outcome and prevailing steroid levels will be a causal function of follicle size.

In this study, we examined the molar concentrations and total content of all three steroids in individual follicles. Although most of the steroids are bound to steroid-binding proteins, they are freely released in immunoassays; as such binding has much low affinity than that of an antibody which at equilibrium obliterates the steroid protein-binding effects [41]. Therefore, the levels of three steroid hormones detected in this study reflect the total amounts of steroids. Results showed that estradiol concentration decreased (figure 2a) while its total content increased significantly with follicle size (figure 2b). Both testosterone and progesterone concentrations remained relatively constant (figure 3a, 4a), while their total content increased (figure 3b, 4b). Thus individual steroid levels within a follicle are a function of follicular volume. Furthermore, no significant association between steroid levels and oocyte recovery or fertilization rates was found. Interestingly, fertilization rates increased dramatically from 30% for follicles not more than 13 mm to 62% for those between 14- 17 mm. Thereafter the fertilization rate slowly declined to 40% for those > 25 mm (see table 2). This suggests a post-maturity phenomenon, and thus overall this study failed to show a simple linear correlation between fertilization rates and follicular diameter. This actually further demonstrates the dominance of follicular size in any physiological relationship. This is inconsistent with a study carried out by Haines and Emes, who reported that once a follicle has reached 10-12 mm in diameter, the fertilization rate remained relatively constant despite progressive follicle growth [21]. This study has concentrated on follicles greater than 10 mm and the size of the study group would give strength to the argument that optimal follicular/oocyte maturity is 14-21 mm.

## Conclusions

For stimulated cycles, luteinized pre-ovulatory follicular fluid levels of testosterone, progesterone and estradiol do not correlate with oocyte maturity (recovery and fertilization rate). Although oocyte fertilization rates correlate with follicular size up to ~21 mm, after this the rate declines, suggesting oocyte post-maturity. In post-luteinized follicles, the steroidogenic ability of granulosa cells is altered to become the dominant cell type producing progesterone. However, these studies do suggest that the oocyte has an influence on this metabolic switching: Although progesterone is the major *de novo *synthesised steroid of luteinised granulosa; when an oocyte is present follicular estradiol correlates with progesterone levels. However, if an oocyte *is not *present follicular estradiol levels correlate with testosterone and not progesterone levels.

## Competing interests

The authors declare that they have no competing interests.

## Authors' contributions

XSW and DL carried out the research and collected the data. AJT and SMD proposed the study and collected the samples. XSW and RKI performed data analysis, interpreted data. XSW drafted the manuscript, RKI and SMD made significant editing for the manuscript. All authors have read and approved the final manuscript.
